# Structural Control of Stromal‐Immune Coupling by *COL24A1* Underlies Pregnancy Maintenance

**DOI:** 10.1002/advs.78110

**Published:** 2026-10-04

**Authors:** Teng Wu, Lingling Xie, Wenyi Liu, Hanjie Mo, Chengshu Xie, Jing Tang, Zhaorui Wang, Peixuan Lan, Jianping Zhang, Jie Gao, Qionglan Tang, Chunwei Cao, Hui Chen

**Affiliations:** ^1^ Department of Obstetrics and Gynecology Sun Yat‐sen Memorial Hospital Sun Yat‐sen University Guangzhou Guangdong China; ^2^ Guangzhou National Laboratory Guangzhou Guangdong China; ^3^ Center of Reproductive Medicine Department of Gynecology & Obstetrics Nanfang Hospital Southern Medical University Guangzhou Guangdong China; ^4^ The First Affiliated Hospital of Guangzhou University of Chinese Medicine Guangzhou China; ^5^ Guangdong Clinical Research Academy of Chinese Medicine Guangzhou China; ^6^ State Key Laboratory of Traditional Chinese Medicine Syndrome Guangzhou China; ^7^ Department of Pathology Sun Yat‐sen Memorial Hospital Sun Yat‐Sen University Guangzhou Guangdong China; ^8^ Guangdong Provincial Key Laboratory of Malignant Tumor Epigenetics and Gene Regulation Guangdong‐Hong Kong Joint Laboratory for RNA Medicine Medical Research Center Sun Yat‐Sen Memorial Hospital Sun Yat‐Sen University Guangzhou Guangdong China

**Keywords:** COL24a1, collagen, extracellular matrix, maternal‐fetal medicine, recurrent pregnancy loss, stromal‐immune crosstalk

## Abstract

Successful pregnancy requires dynamic extracellular matrix (ECM) remodeling at the maternal‐fetal interface, yet whether individual collagen subtypes actively regulate this process remains unknown. Here, collagen type XXIV alpha 1 chain (COL24A1), a uterine‐enriched fibrillar collagen, is identified as a regulator of pregnancy‐associated ECM‐immune homeostasis. *Col24a1*‐deficient mice exhibit increased embryo resorption, fetal growth impairment, and pregnancy‐dependent uterine collagen accumulation and inflammation. Mechanistically, *Col24a1* loss promotes expansion of a maladaptive *Timp1*‐high stromal state, accompanied by reduced matrix metalloproteinase 2 activity, impaired ECM degradation, and altered stromal‐immune communication and inflammatory immune‐cell states. *COL24A1* silencing in human endometrial stromal cells similarly impairs decidualization and increases *TIMP1* and fibrillar collagen gene expression. Hydroxychloroquine partially rescues embryo resorption and attenuates uterine inflammation, indicating that these pregnancy‐associated abnormalities are pharmacologically modifiable. Moreover, a patient with 12 consecutive unexplained early pregnancy losses carrying rare heterozygous *COL24A1* variants shows a concordant decidual phenotype with reduced COL24A1 and increased TIMP1, inflammation, and collagen deposition. These findings identify *COL24A1* as a collagen subtype‐specific regulator of maternal‐fetal ECM‐immune homeostasis and nominate it as a candidate susceptibility gene for unexplained recurrent pregnancy loss.

## Graphical abstract

COL24A1 emerges as a collagen‐associated regulator of maternal‐fetal interface homeostasis. Its deficiency promotes a maladaptive TIMP1‐high stromal state, impaired ECM turnover, collagen accumulation, and stromal–immune dysregulation, contributing to pregnancy loss. Partial rescue by hydroxychloroquine and a concordant decidual phenotype in a patient carrying *COL24A1* variants support the potential translational relevance of this ECM‐immune axis. (The Graphical Abstract was created with assistance from Gemini 3 Pro Image (Google; used February 2026) for visual illustration. Its scientific content was defined by the authors, and the AI‐assisted output was reviewed and revised by the authors for accuracy).

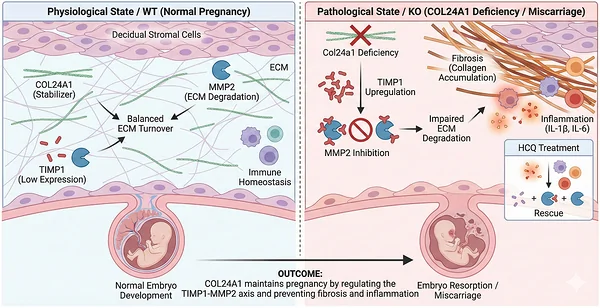

**Abbreviations**
COL24A1: Collagen type XXIV alpha 1 chain
ECM: Extracellular matrix
KO: Knockout
GD13.5: Gestational day 13.5
HCQ: Hydroxychloroquine
IL‐1β: Interleukin 1 beta
IL‐6: Interleukin 6
MMPs: Matrix metalloproteinases
RPL: Recurrent pregnancy loss
SNP: Single nucleotide polymorphism
TIMP1: Tissue inhibitor of metalloproteinase 1
WT: Wild type

## 1 Introduction

Recurrent pregnancy loss (RPL), defined as two or more failed clinical pregnancies before 20–24 weeks of gestation, affects ∼1%‐5% of couples attempting to conceive and carries substantial psychological and physical burdens [1, 2]. Despite standardized endocrine, cytogenetic, and uterine evaluations, the etiology remains unidentified in approximately half of cases, leaving unexplained RPL without mechanism‐based diagnostics or targeted therapy [2, 3].

Early pregnancy success requires a receptive endometrium and tightly coordinated cellular programs at the maternal‐fetal interface [4, 5, 6]. Beyond a structural scaffold, the decidual extracellular matrix (ECM) provides biochemical and biomechanical signals that shape stromal differentiation, regulate trophoblast invasion, and influence immune‐cell positioning and tissue adaptation [4, 5, 7, 8]. Extensive ECM remodeling, including dynamic changes in collagen composition and organization, is a well‐established feature of decidualization in rodents and human endometrium [9, 10, 11]. As dominant ECM constituents, collagens organize matrix architecture and turnover; however, mechanistic work has largely centered on major fibrillar collagens (e.g., types I and III) [7, 12, 13], while many fibril‐associated or non‐fibrillar collagen isoforms remain poorly defined in decidual biology, representing a key gap for unexplained RPL.

The *COL24A1* gene, located on chromosome 1, encodes the collagen type XXIV α1 chain, a large fibril‐associated collagen comprising 1,714 amino acids [14] and primarily characterized in developmental contexts [15]. Emerging human genetic data revealing that the maternal SNP rs11161721 synergizes with pre‐pregnancy obesity to elevate preterm birth risk [16] now thrust it onto the pregnancy stage, while its robust expression in uterine fibroids hints at active participation in ECM remodeling [17]. Yet whether COL24A1 is required for pregnancy maintenance and how it contributes to maternal‐fetal interface homeostasis remain unknown.

Here, by integrating a *Col24a1*‐null mouse model with bulk and single‐cell transcriptomics, pharmacological rescue, and human evidence from a patient with idiopathic RPL, we identify *COL24A1* deficiency as a driver of maternal‐fetal interface dysfunction. Loss of *COL24A1* reshapes uterine stromal cell states towards a maladaptive phenotype characterized by impaired ECM remodeling and altered stromal‐immune communication. These findings nominate *COL24A1* as a candidate susceptibility gene for unexplained RPL and implicate stromal‐immune dysregulation as a potentially tractable mechanism underlying pregnancy failure.

## 2 Methods

### 2.1 Study Approval

Human study protocols were approved by the ethics committee of Sun Yat‐sen Memorial Hospital, Sun Yat‐sen University (2017‐BA‐04), and all participants provided written informed consent. Animal studies were approved by the Animal Care and Ethics Committee of Sun Yat‐sen University (SYSU‐IACUC‐2022‐001356 and SYSU‐IACUC‐2022‐001220), and were conducted in accordance with the National Institutes of Health Guidelines on the Care and Use of Animals.

### 2.2 Statistical Analysis

Data are presented as mean ± SEM unless otherwise specified. Statistical analyses were performed in GraphPad Prism (v8.4.3; GraphPad Software). Normality was assessed using the Shapiro‐Wilk test. For normally distributed data, two‐group comparisons used a two‐tailed unpaired Student's t test, with Welch's correction when variances were unequal. Multiple‐group comparisons used one‐way or two‐way ANOVA, followed by the appropriate post hoc test (eg, Dunnett's or Tukey's). For non‐normally distributed data, the Mann‐Whitney U test (two groups) or Kruskal‐Wallis test (>2 groups) with Dunn's post hoc test was used. A two‐sided *p* < 0.05 was considered statistically significant. In figures, significance is denoted as ^*^p <0.05, ^**^p <0.01, and ***p <0.001.

## 3 Results

### 3.1 *Col24a1* Is Highly Expressed in the Uterus

COL24A1 encodes collagen XXIV, a fibrillar collagen component of the extracellular matrix (ECM). Analysis of the Expression Atlas database revealed relatively high uterine COL24A1/Col24a1 expression in the uterus of humans, rats, and mice compared to other tissues (Figure 1A). qPCR analysis of tissue expression profiles further confirmed high *Col24a1* expression in the uterus of both mice and rats (Figure 1B,C). Additionally, the collagenous “Gly‐X‐Y” repeat region of COL24A1 was highly conserved across humans, rats, and mice (Figure 1D). Together, these data indicate that *Col24a1* exhibits relatively high uterine expression and suggest a potential role in female reproductive function.

**FIGURE 1:**
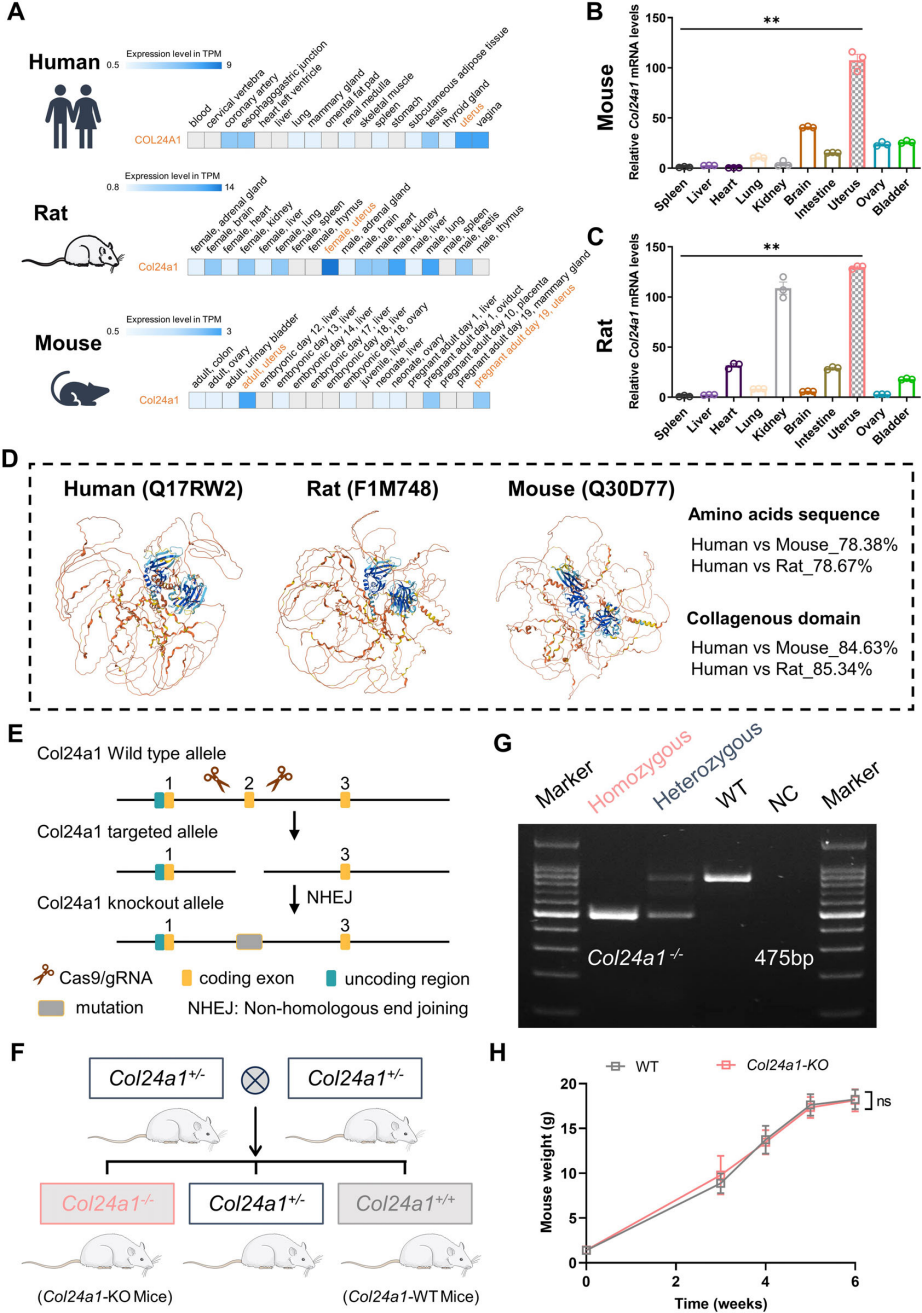
COL24A1 is highly expressed in the uterus, and *Col24a1* knockout mice were generated. (A) Baseline expression of COL24A1 across human, rat, and mouse tissues, demonstrating uterus‐enriched expression (EMBL‐EBI Expression Atlas; baseline dataset). (B) Relative *Col24a1* mRNA levels in the indicated mouse tissues measured by RT‐qPCR (n = 3 mice per group). (C) Relative *Col24a1* mRNA levels in the indicated rat tissues measured by RT‐qPCR (n = 3 rats per group). (D) Schematic comparison of predicted COL24A1 protein architectures in human, rat, and mouse. (E) CRISPR/Cas9 strategy for generating *Col24a1* knockout mice by deleting exon 2 to disrupt COL24A1 expression. (F) Breeding scheme to obtain *Col24a1* heterozygous and homozygous knockout offspring. (G) Representative PCR genotyping identifying wild‐type, heterozygous, and homozygous knockout mice using allele‐specific amplicons. (H) Body weight growth curves of female wild‐type (WT; n = 12) and *Col24a1* knockout (KO; n = 24) mice. Statistical analysis was performed by the non‐parametric Kruskal‐Wallis test followed by Dunn's post‐hoc test (B, C) and two‐way ANOVA followed by Bonferroni's multiple comparisons test (H). Data are mean ± SEM. Differences were indicated as statistically significant at ^**^p <0.01.

### 3.2 *Col24a1* Knockout Induces Embryo Loss in Mice

Given that *Col24a1* showed relatively high expression in the mouse uterus, we investigated the effects of *Col24a1* loss of function on reproductive outcomes. To this end, we generated a *Col24a1* knockout (*Col24a1‐KO*) mouse model using CRISPR/Cas9 (Figure 1E–G and Figure S1A,B). *Col24a1‐KO* mice exhibited no obvious gross abnormalities and showed no significant differences in body weight compared with wild‐type (WT) mice (Figure 1H). Female WT and *Col24a1*‐*KO* mice were subsequently mated with healthy WT males and euthanized at gestational day 13.5 (GD13.5) (Figure 2A). *Col24a1‐KO* mice exhibited a significantly higher embryo resorption rate than WT controls (Figure 2B,C). Notably, age‐stratified analysis showed that the increased embryo resorption associated with *Col24a1* deficiency was already evident in younger females and was further exacerbated in older females (Figure S2A,B). In addition, *Col24a1‐KO* mice showed a reduced number of embryos per dam and lower fetal and placental weights (Figure 2D–F). Further analysis revealed no significant differences in serum reproductive hormone levels between WT and *Col24a1*‐*KO* mice in the non‐pregnancy state or during pregnancy (Figure 2G–I). Likewise, no significant alterations in estrous cyclicity were observed between the two groups (Figure 2J). These findings indicate that the pregnancy phenotype associated with *Col24a1* deficiency was not accompanied by major alterations in the measured serum reproductive hormone levels or estrous cyclicity. Taken together, these results demonstrate that *Col24a1* loss compromises pregnancy maintenance and promotes embryo loss.

**FIGURE 2:**
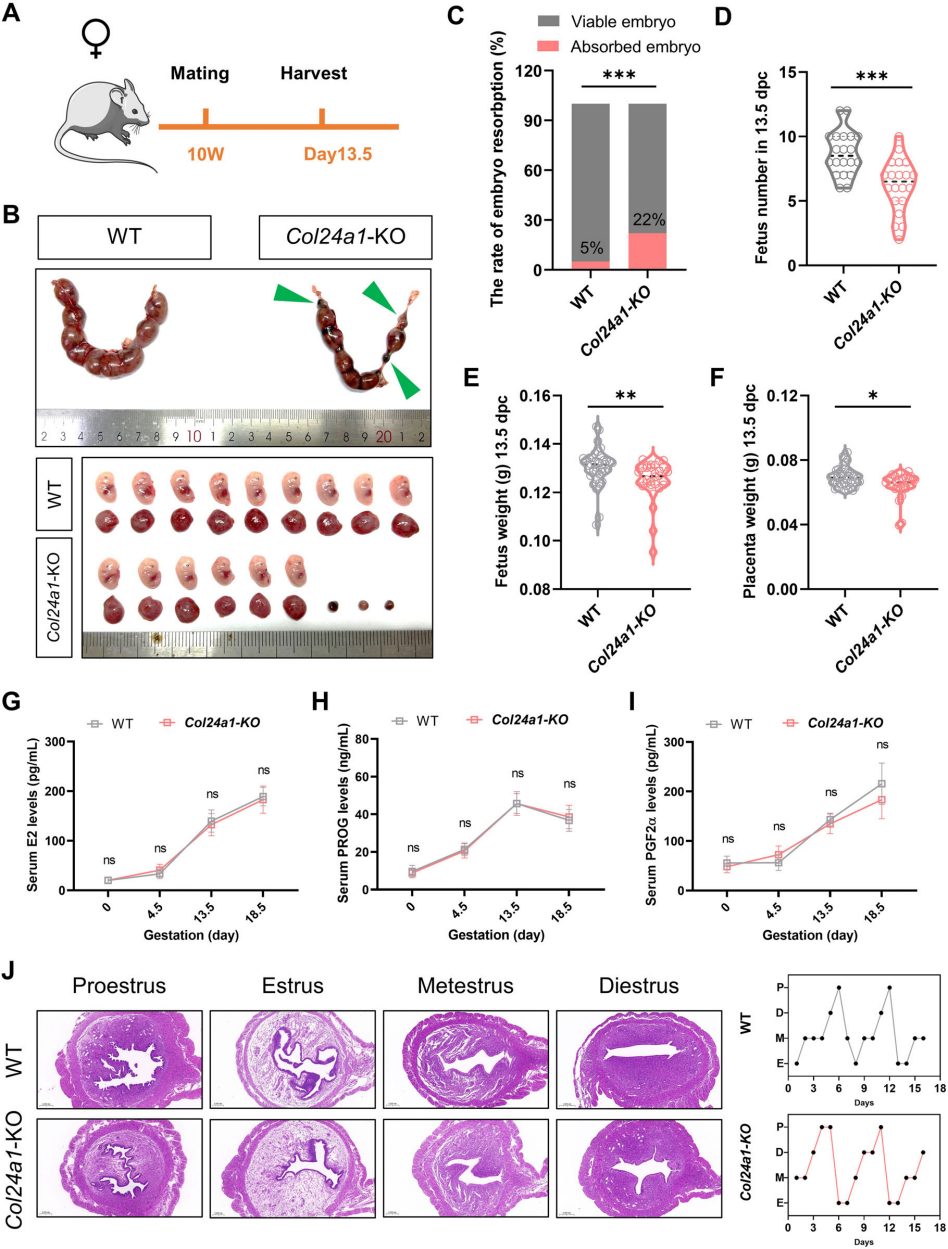
*Col24a1* deficiency induces pregnancy loss in mice. (A) Schematic of the pregnancy study design and sampling timeline. (B) Representative gross morphology of uteri and the corresponding embryos and placentas at GD13.5. Green arrows indicate resorbed implantation sites with marked uterine hemorrhage. (C) Embryo resorption rate at GD13.5 in wild‐type (WT) and *Col24a1* knockout (KO) dams. (D) Number of embryos per dam at GD13.5 in WT and KO groups (n = 20 pregnancies per group). (E) Individual fetal weight and (F) individual placental weight at the indicated gestational stages (four litters per group). (G–I) Serum estradiol (E2; G), progesterone (PROG; H), and prostaglandin F2α (PGF2α; I) concentrations in WT and KO mice at the indicated gestational stages. (J) Estrous cycle profiles in WT and KO females. Scale bars, 200 µm. (D, E, F) Violin plots show lines at the median (black dashed) and quartiles (dashed). Statistical analysis was performed by Chi‐square test (C), unpaired Student's t‐test (D), non‐parametric Mann‐Whitney test (E, F), and two‐way ANOVA followed by Bonferroni's multiple comparisons test (G–I). Data are mean ± SEM. Differences were indicated as statistically significant at ^*^p <0.05, ^**^p < 0.01, and ^***^p <0.001.

### 3.3 *Col24a1* Knockout Promotes Uterine Collagen Deposition and Inflammation During Pregnancy

To investigate the pathological changes associated with *Col24a1* knockout‐induced pregnancy loss in mice, we further examined collagen deposition and inflammation in uterine tissue. Histopathological analysis revealed significantly increased uterine collagen deposition in *Col24a1* knockout mice compared with WT mice at GD 13.5 (Figure 3A). Hydroxyproline assays further confirmed the increase in total uterine collagen content (Figure 3B). Transmission electron microscopy revealed that uterine collagen fibrils in *Col24a1* knockout mice were more densely packed and less regularly organized than those in WT mice at GD13.5 (Figure 3C). At the molecular level, key collagen and inflammatory markers were examined in uterine tissue. Immunoblotting showed that *Col24a1* knockout significantly increased the protein levels of COL1a1, COL3a1, IL‐6, and IL‐1β at GD 13.5 compared with WT controls (Figure 3D). Immunohistochemistry further showed increased COL1a1 and IL‐1β staining in the uteri of *Col24a1* knockout mice at GD 13.5 (Figure 3E,F).

**FIGURE 3:**
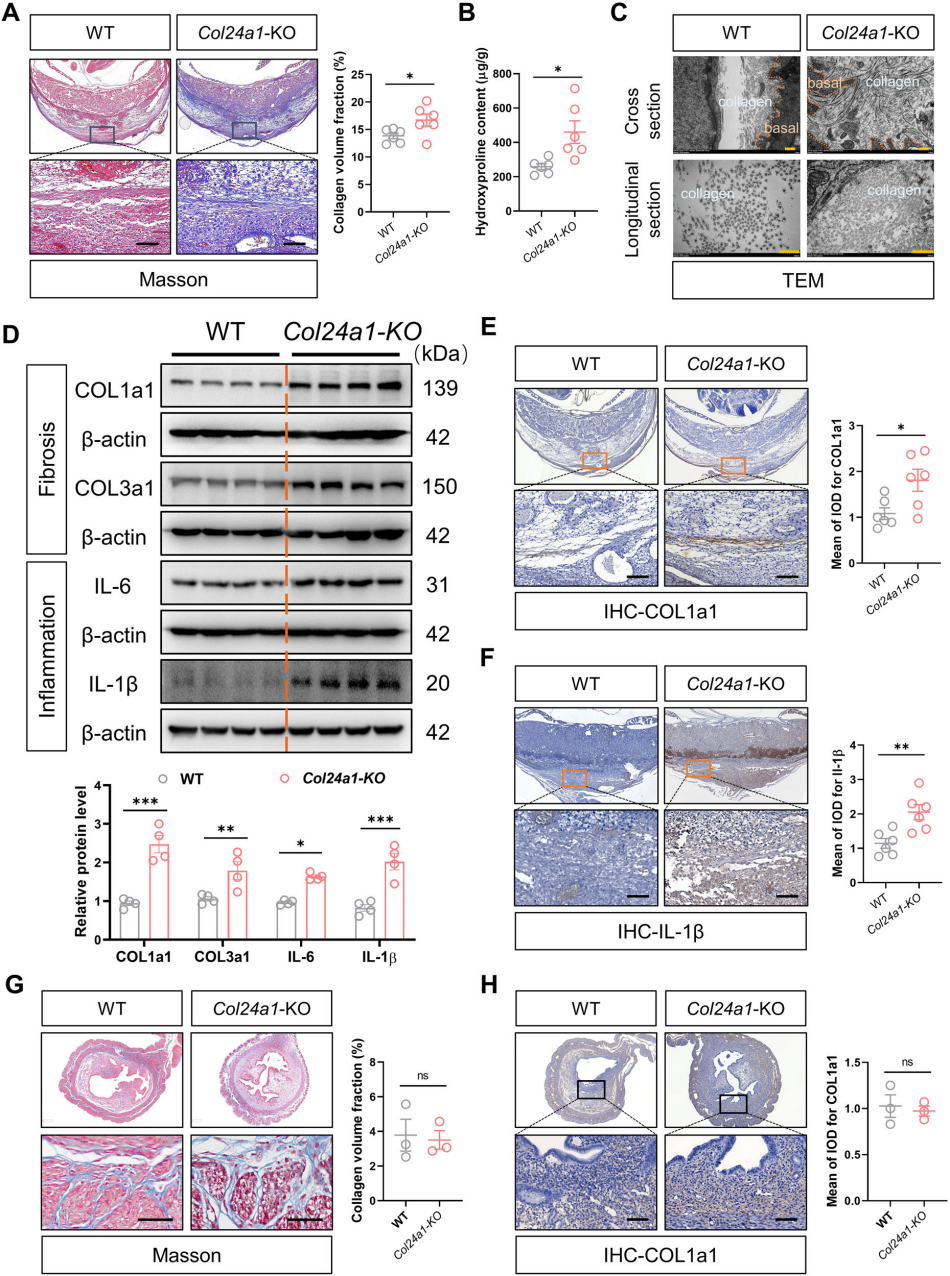
*Col24a1* deficiency increases uterine collagen deposition and inflammation during pregnancy. (A) Representative Masson's trichrome staining of uterine sections at GD13.5 and quantification of collagen‐positive area. Collagen‐positive area was quantified as a percentage of the tissue area using NIH ImageJ. n = 6 mice per group. Scale bars, 100 µm. (B) Comparison of hydroxyproline content in uterine tissues from WT and *Col24a1‐KO* mice at GD13.5 (n = 6 mice per group). (C) Representative transmission electron microscopy images of the basal stromal region of the uterine wall at GD13.5. The basal region and collagen‐rich areas are indicated in the images. Collagen fibrils are shown in transverse (top) and longitudinal (bottom) orientations. Scale bars: 500 nm. (D) Representative immunoblots and densitometric analyses of COL1a1, COL3a1, IL‐1β, and IL‐6 in uterine tissues from WT (n = 4) and *Col24a1* knockout mice (n = 4) at GD13.5; β‐actin served as the loading control. (E, F) Representative immunohistochemistry (IHC) staining and quantification of COL1a1 (E; brown) and IL‐1β (F; brown) in uteri at GD13.5 (n = 6 mice per group). Nuclei were counterstained with hematoxylin (blue). Scale bars, 100 µm. (G) Representative Masson's trichrome staining of uterine sections from nonpregnant WT and *Col24a1*‐*KO* mice, with quantification of collagen‐positive area (n = 3 mice per group). Scale bars, 50 µm. (H) Representative IHC staining and quantification of COL1a1 (brown) in nonpregnant uterine sections (n = 3 mice per group). Nuclei were counterstained with hematoxylin (blue). Scale bars, 100 µm. Statistical analysis was performed by unpaired Student's t‐test (A, B, E, F), two‐way ANOVA followed by Bonferroni's multiple comparisons test (D), and non‐parametric Mann‐Whitney test (G, H). Data are mean ± SEM. Differences were indicated as statistically significant at ^*^p <0.05, ^**^p < 0.01, and ^***^p <0.001.

To determine whether these pathological changes were also present in the nonpregnant state, we further examined uterine tissues from nonpregnant WT and *Col24a1* knockout mice. Masson's trichrome staining revealed no significant difference in uterine collagen deposition between the two groups (Figure 3G). Similarly, immunoblotting showed no significant differences in the protein levels of COL1a1, COL3a1, IL‐6, or IL‐1β between WT and *Col24a1* knockout uteri in the nonpregnant state (Figure S2C). Immunohistochemical analysis further showed no significant difference in uterine COL1a1 staining between the two groups (Figure 3H). These data indicate that *Col24a1* deficiency does not produce an overt fibrotic or inflammatory uterine phenotype under nonpregnant conditions.

Collectively, these findings indicate that *Col24a1* deficiency is associated with pregnancy‐dependent increases in uterine collagen deposition and inflammation, which may contribute to the adverse pregnancy phenotype observed in *Col24a1* knockout mice.

### 3.4 *Col24a1* Knockout Upregulates TIMP1 and Suppresses Uterine Extracellular Matrix Degradation During Pregnancy

Given that *Col24a1* knockout induces pregnancy loss in mice, we sought to investigate the underlying molecular mechanisms. To this end, we performed bulk RNA sequencing of uterine tissues from WT and *Col24a1* knockout mice at GD13.5 (Figure S3A). PCA showed that samples from the two groups clustered separately (Figure 4A), indicating distinct global transcriptional profiles between WT and *Col24a1* knockout uteri. Differential expression analysis identified 452 differentially expressed genes (DEGs) in *Col24a1* knockout uteri compared with WT controls, including 295 upregulated and 157 downregulated genes (Figure 4B). Functional enrichment analysis showed that the downregulated DEGs were mainly enriched in “Negative regulation of cell adhesion” and “Degradation of the extracellular matrix” (Figure 4C). Consistently, GSEA further showed enhancement of cell‐cell adhesion and suppression of ECM degradation (Figure S3B). Furthermore, the upregulated DEGs were mainly enriched in “positive regulation of cell‐substrate adhesion” and “positive regulation of cell junction assembly” (Figure S3C).

**FIGURE 4:**
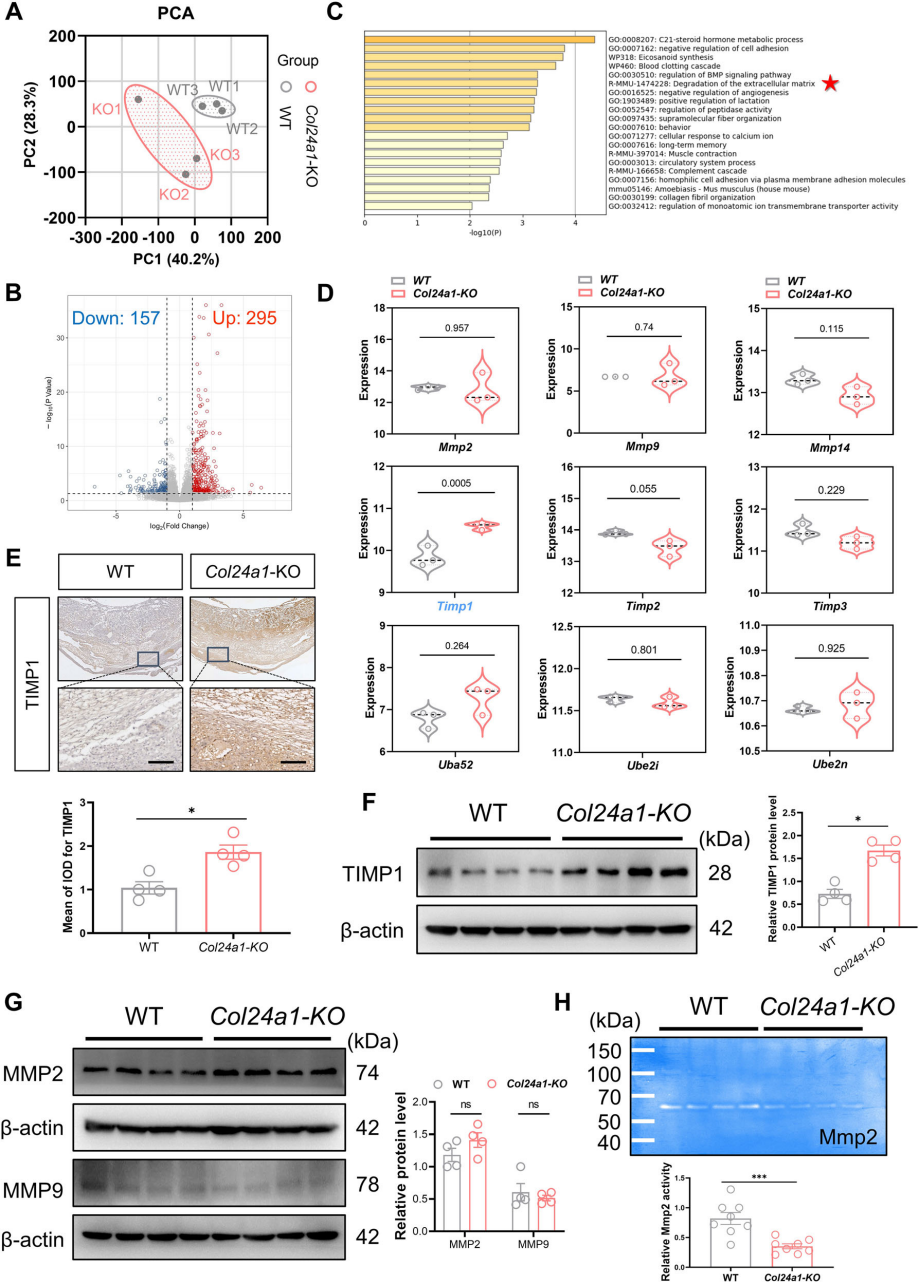
*Col24a1* knockout upregulates uterine TIMP1 and suppresses extracellular matrix degradation during pregnancy. (A) Principal component analysis (PCA) of uterine transcriptomes at GD13.5 shows separation between wild‐type (WT) and *Col24a1* knockout (KO) groups. (B) Volcano plot of differentially expressed genes in KO versus WT uterine tissues at GD13.5. (C) Gene Ontology (GO) enrichment analysis of genes significantly downregulated in KO uteri compared with WT controls. (D) Violin plots of genes involved in extracellular matrix (ECM) degradation in WT and KO uteri at GD13.5. (E) Representative immunohistochemistry (IHC) staining (brown) and quantification of TIMP1 in uterine sections at GD13.5 (n = 4 mice per group); nuclei were counterstained with hematoxylin (blue). Scale bars, 100 µm. (F) Representative immunoblots and densitometric quantification of TIMP1 in uterine tissues at GD13.5. (G) Representative immunoblots and densitometric analyses of MMP2 and MMP9 in uterine tissues at GD13.5. (H) Representative gelatin zymography and quantification of MMP2 activity in uterine tissues at GD13.5 (n = 8 mice per group). Statistical analysis was performed by two‐sided Wilcoxon rank‐sum test with Bonferroni correction (D), non‐parametric Mann‐Whitney test (E, F), two‐way ANOVA followed by Bonferroni's multiple comparisons test (G), and unpaired Student t test (H). Data are mean ± SEM. Differences were indicated as statistically significant at ^*^p <0.05, ^**^p < 0.01, and ^***^p <0.001.

Based on the observation that *Col24a1* knockout promotes uterine collagen deposition during pregnancy (Figure 3), we hypothesized that *Col24a1* deficiency may contribute to collagen accumulation by impairing ECM degradation. We therefore examined key components involved in ECM turnover, including matrix metalloproteinases (MMPs), tissue inhibitors of metalloproteinases (TIMPs), and ubiquitin‐proteasome‐related genes. The analysis revealed no significant differences in the expression of *Mmp2*, *Mmp9*, *Mmp14*, *Uba52*, *Ube2i*, or *Ube2n* between WT and *Col24a1* knockout uteri (Figure 4D). Notably, *Timp1* was significantly upregulated in *Col24a1* knockout uteri compared with WT controls (Figure 4D). Immunohistochemistry (Figure 4E) and immunoblotting (Figure 4F) further confirmed increased TIMP1 protein levels in *Col24a1* knockout uteri at GD 13.5. In contrast, TIMP1 protein levels did not differ significantly between WT and *Col24a1* knockout uteri in the nonpregnancy state (Figure S3D).

TIMP1 inhibits the activity of multiple MMPs by interacting with their catalytic domains and has important roles in physiological processes including pregnancy, embryonic development, angiogenesis, and wound healing [18]. Immunoblotting revealed no significant differences in MMP2 and MMP9 protein levels between WT and *Col24a1* knockout uteri at GD 13.5 (Figure 4G). However, gelatin zymography showed that MMP2 activity was significantly reduced in *Col24a1* knockout uteri compared with WT controls, whereas MMP9 activity was unchanged (Figure 4H).

Taken together, these findings indicate that *Col24a1* deficiency is associated with increased TIMP1 expression in the uterus, reduced MMP2 activity, and impaired ECM degradation during pregnancy. These alterations may contribute to excessive collagen deposition in *Col24a1*‐knockout uteri.

### 3.5 ScRNA‐seq Reveals That Col24a1 May Act as a Buffering Factor That Maintains Homeostasis at the Maternal‐Fetal Interface During Pregnancy

To characterize the early cellular changes associated with *Col24a1* deficiency and pregnancy loss, we performed single‐cell RNA sequencing (scRNA‐seq) of uterine tissues from WT and *Col24a1‐KO* mice at GD 5.5 (Figure S4A). Based on canonical marker genes and cluster‐specific expression patterns, we identified 19 major uterine cell types, including stromal, immune, epithelial, endothelial, smooth muscle, pericyte, and mesothelial populations (Figure 5A,B and Figure S4B,C). *Col24a1* transcripts were preferentially expressed in stromal population 3 (Str_3_Tenm3), whereas *Timp1*, which was also identified in our bulk RNA‐seq analysis, was predominantly expressed in stromal population 4 (Str_4_Clec3b) (Figure 5C). Notably, *Col24a1‐KO* uteri exhibited a disproportionate expansion of str_4_Clec3b relative to WT uteri (Figure 5B), implicating this subset as a potential contributor to early pathological alterations in the uterine microenvironment.

**FIGURE 5:**
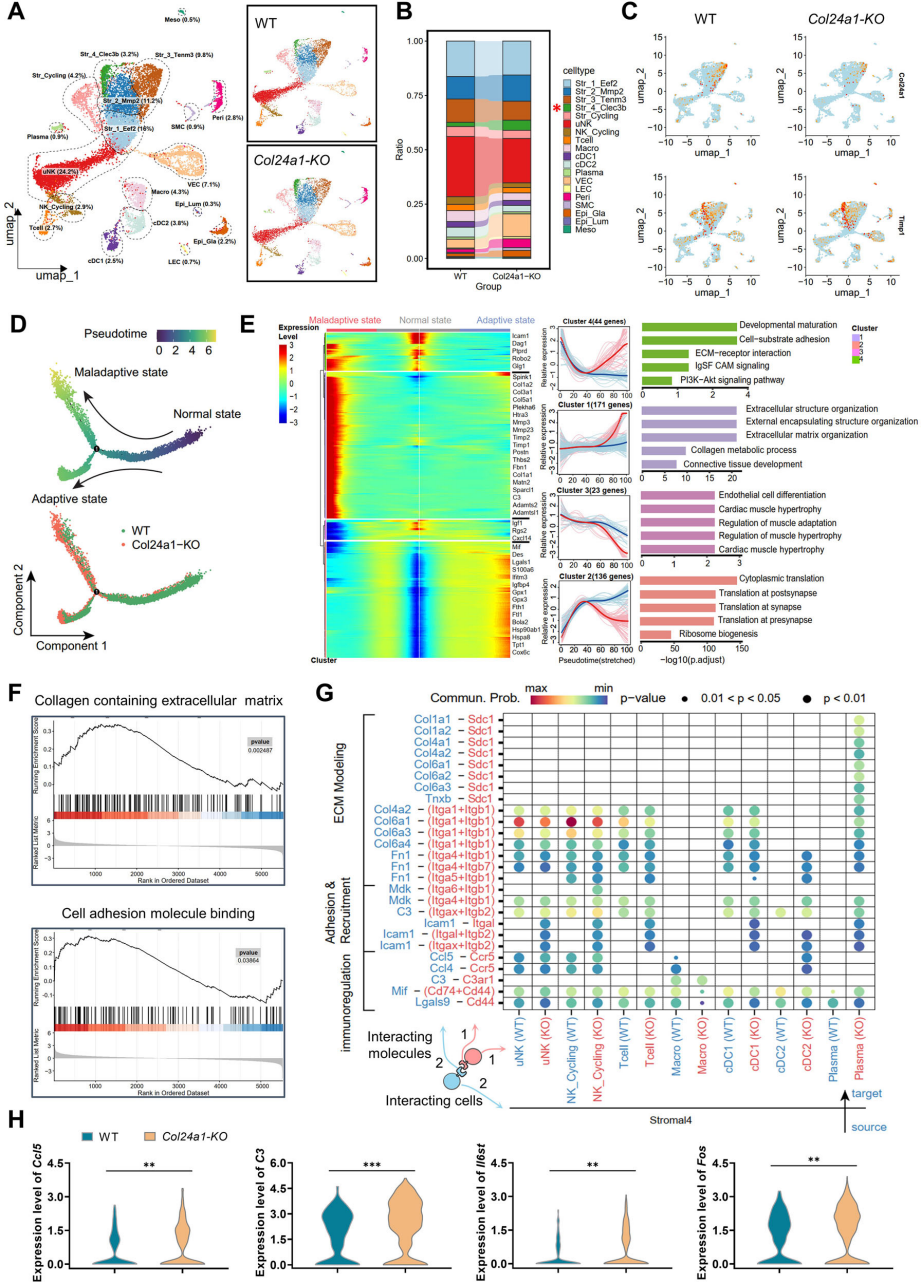
Overview of the single‐cell transcriptional landscape of uterine tissues from WT and *Col24a1‐KO* mice. (A) Dimensionality reduction of scRNA‐seq data using uniform manifold approximation and projection (UMAP), comparing cell clusters between WT and *Col24a1‐KO* mice. cDC, conventional dendritic cells; Epi_Gla, glandular epithelium; Epi_Lum, luminal epithelium; LEC, lymphatic endothelial cells; Macro, macrophages; Meso, mesothelium; Peri, pericytes; SMC, smooth muscle cells; Str, stromal cells; uNK, uterine natural killer cells; VEC, vascular endothelial cells. (B) Comparison of cell type proportions between WT and *Col24a1‐KO* mice. (C) UMAP plots showing *Col24a1* and *Timp1* expression in uterine cells from WT and *Col24a1*‐KO mice. (D) Monocle analysis showing the ordering of stromal cells along pseudotime, colored by cell state. (E) Heatmap showing four gene clusters with distinct dynamic expression patterns along the pseudotime trajectory (left). Representative cell adhesion‐related and collagen metabolism‐related genes are labeled. Gene Ontology biological process enrichment analysis of the gene clusters is shown on the right. (F) GSEA showing enrichment of collagen‐containing extracellular matrix and cell adhesion pathways in the Stromal 4 subpopulation of *Col24a1‐KO* mice compared with WT controls. (G) Overview of selected ligand‐receptor interactions; *p*‐values are represented by circle size (scale at top), and the mean expression levels of the interacting molecules in the corresponding cell populations are indicated by color. The interactions were inferred from transcriptomic data and represent predicted ligand‐receptor interactions. (H) Violin plots showing the expression of inflammation‐related genes in the Stromal 4 subpopulation across groups.

To infer stromal‐state dynamics associated with *Col24a1* deficiency, we integrated stromal cells from WT and *Col24a1‐KO* uteri and reconstructed pseudotime trajectories. This analysis revealed distinct state distributions between the two genotypes (Figure 5D and Figure S4D). Stromal cells from *Col24a1‐ KO* uteri were distributed across two representative transcriptional states, designated adaptive and maladaptive states (Figure 5D), suggesting that *Col24a1* loss alters stromal‐state dynamics during early pregnancy. Genes exhibiting similar dynamic expression patterns along the pseudotime trajectories were subsequently grouped into four gene clusters (clusters 1–4) (Figure 5E). Compared with the adaptive state, the maladaptive state was characterized by increased expression of fibrillar collagen genes, including *Col1a1* and *Col3a1*, together with adhesion‐associated and matrix‐interacting genes such as *Icam1*, *Dag1*, and *Htra3* (Figure 5E). Consistently, Str_4_Clec3b cells were preferentially distributed along the maladaptive branch (Figure S4D), in agreement with the histological phenotype of increased collagen accumulation and suggesting an association of this stromal subset with altered ECM remodeling and adhesion programs. Gene set enrichment analysis further showed enrichment of collagen‐containing extracellular matrix and cell adhesion molecule binding pathways in Str_4_Clec3b cells (Figure 5F).

Given that the expansion of the maladaptive Str_4 population was associated with altered ECM and cell adhesion programs, we hypothesized that this stromal shift may disturb stromal‐immune coordination at the maternal‐fetal interface. We therefore examined predicted intercellular communication between Str_4 cells and immune populations (Figure 5G). CellChat‐based inference suggested extensive alterations in ligand‐receptor communication in *Col24a1‐KO* uteri, involving signaling programs related to ECM remodeling, adhesion and cellular recruitment, and immunomodulation (Figure 5G). ECM remodeling‐ and adhesion‐associated networks involving collagen, tenascin XB (*Tnxb*), fibronectin 1 (*Fn1*), midkine (*Mdk*), complement C3 (*C3*), and intercellular adhesion molecule 1 (*Icam1*) were prominent and predicted to originate predominantly from Str_4 cells. These signals were predicted to engage immune populations expressing syndecan 1 (*Sdc1*) and multiple integrin receptors, consistent with altered ECM‐receptor communication that may influence immune‐cell adhesion, migration, and spatial organization (Figure 5G). In parallel, immunoregulatory signaling involving C‐C motif chemokine ligands (*Ccls*), complement components, macrophage migration inhibitory factor (*Mif*), galectin 9 (*Lgals9*), C‐C motif chemokine receptor 5 (*Ccr5*), *Cd74*, and *Cd44* formed an altered communication network, suggesting altered immune recruitment and activation states at this stage of gestation (Figure 5G). Consistently, genes associated with inflammation, ECM organization, and adhesion were expressed at higher levels in Str_4 cells from *Col24a1‐KO* uteri than in WT controls (Figure 5H and Figure S4E).

We further quantified the relative abundances of major immune‐cell populations, including uNK cells, T cells, macrophages, dendritic cells, and plasma cells. Unexpectedly, their relative proportions were either reduced or largely unchanged in *Col24a1‐KO* uteri compared with WT controls (Figure 5B). Given that uNK cells are the predominant immune population at the maternal‐fetal interface and play critical roles in decidualization and pregnancy maintenance, we further examined their heterogeneity and identified five transcriptionally distinct uNK states: Gzma^+^Cma1^+^ effector uNK, Gzmc^+^Lgals3^+^ inflammation‐associated uNK, Tcf7^+^Tcf12^+^ immature uNK, Klra7^+^Cd2^+^ resting uNK, and Stmn1^+^Birc5^+^ cycling uNK (Figure S5A–C). Notably, the relative proportion of Gzmc^+^Lgals3^+^ inflammation‐associated uNK cells was increased in *Col24a1‐KO* uteri, accompanied by increased expression of inflammatory genes and stronger inflammatory‐response signatures (Figure S5D–F).

Collectively, these findings indicate that *Col24a1* contributes to maintaining stromal homeostasis during early pregnancy. *Col24a1* deficiency was associated with expansion of a maladaptive stromal subset characterized by altered ECM remodeling and adhesion programs, accompanied by changes in stromal‐immune communication and immune cell states rather than a generalized increase in immune cell abundance.

### 3.6 *COL24A1* Silencing Impairs In Vitro Decidualization and Alters ECM‐Associated Gene Expression

To determine the effect of *COL24A1* downregulation on decidualization, we transfected human endometrial stromal cells (HESCs) with *COL24A1*‐targeting siRNAs and subsequently induced decidualization in vitro. *COL24A1* expression was reduced by approximately 70% after siRNA transfection (Figure S6A). On day 6 of decidualization, *COL24A1* knockdown significantly reduced the expression of decidualization markers compared with the siNC control (Figure S6B). Notably, *COL24A1* mRNA levels progressively increased during decidualization in control cells (Figure S6C).

Given that *Col24a1* deficiency was associated with abnormal ECM remodeling in the pregnant uterus, we next examined ECM‐related gene expression during HESC decidualization after *COL24A1* knockdown. *TIMP1* mRNA levels increased during decidualization and were further elevated in *COL24A1*‐silenced cells (Figure S6D). Likewise, *COL1A1* and *COL3A1* mRNA levels increased during decidualization and were further increased following *COL24A1* knockdown (Figure S6E,F).

Together, these findings show that *COL24A1* knockdown impairs HESC decidualization and is accompanied by increased expression of *TIMP1* and fibrillar collagen genes.

### 3.7 Hydroxychloroquine Sulfate Reduced Embryo Resorption in *Col24a1* Knockout Mice

Given that *Col24a1* deficiency was associated with uterine inflammation, fibrosis, and pregnancy loss in mice, we next investigated whether pharmacological intervention could ameliorate the adverse pregnancy outcomes. Hydroxychloroquine (HCQ) is an antimalarial drug with well‐established immunomodulatory and anti‐inflammatory effects and is widely used to treat rheumatic autoimmune diseases, particularly systemic lupus erythematosus [19, 20]. We therefore assessed the effects of HCQ treatment on pregnancy outcomes in *Col24a1*‐deficient mice.

To evaluate the effects of HCQ treatment in vivo, *Col24a1* knockout mice received HCQ‐containing or regular drinking water beginning at 7 weeks of age, were mated with healthy WT males at 8 weeks of age, and were euthanized at GD 13.5 (Figure 6A). HCQ treatment significantly reduced the embryo resorption rate in *Col24a1* knockout mice compared with water‐treated controls and improved fetal and placental growth (Figure 6B–E). Notably, HCQ did not significantly affect pregnancy outcomes in WT mice under the same experimental conditions (Figure S7A–D). In addition, HCQ concentrations were markedly increased in the serum and uterine tissues of HCQ‐treated *Col24a1* knockout mice compared with water‐treated controls, confirming systemic and uterine tissue exposure to the drug (Figure 6F).

**FIGURE 6:**
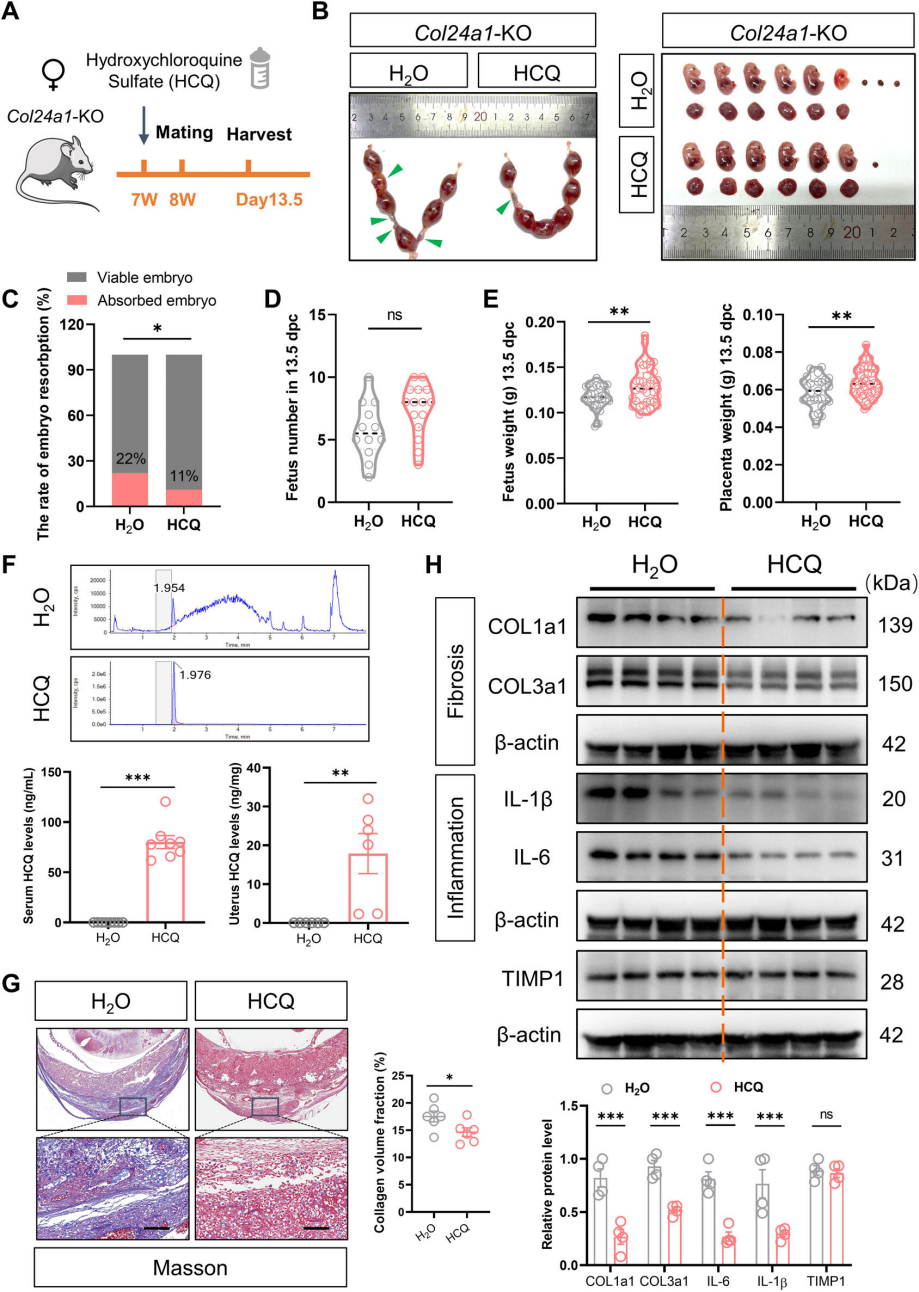
Hydroxychloroquine sulfate reduces embryo resorption in *Col24a1* knockout mice. (A) Schematic of the experimental design, including hydroxychloroquine sulfate (HCQ) administration and the assessment timeline. (B) Representative gross morphology of uteri and the corresponding embryos and placentas at GD13.5. Green arrows indicate resorbed implantation sites. (C) Embryo resorption rate at GD13.5 in the indicated treatment groups. (D) Number of embryos per dam at GD13.5 (n = 12 or 15 dams per group). (E) Fetal and placental weights at GD13.5. (F) Representative mass spectrometry peak for HCQ and quantification of HCQ levels in serum and uterine tissues at GD13.5 (n = 8 or 6 mice per group). (G) Representative Masson's trichrome staining of uterine sections at GD13.5 and quantification of the collagen‐positive area (n = 6 mice per group). Scale bars, 100 µm. (H) Representative immunoblots and densitometric analyses of COL1a1, COL3a1, IL‐1β, IL‐6, and TIMP1 in uterine tissues from H_2_O‐treated (n = 4) and HCQ‐treated (n = 4) mice at GD13.5; β‐actin served as the loading control. Violin plots show lines at the median (black dashed) and quartiles (dashed). Statistical analysis was performed by Chi‐square test (C), unpaired Student's t test (D–G), and two‐way ANOVA followed by Bonferroni's multiple comparisons test (H). Data are mean ± SEM. Differences were indicated as statistically significant at ^*^p <0.05, ^**^p < 0.01, and ^***^p <0.001.

Consistent with its known anti‐inflammatory properties, HCQ treatment significantly reduced uterine IL‐1β expression in *Col24a1*‐deficient mice at GD13.5 (Figure S7E). Masson's trichrome staining further showed reduced uterine collagen deposition after HCQ treatment (Figure 6G). Immunoblotting demonstrated reduced levels of collagen‐ and inflammation‐associated proteins in HCQ‐treated *Col24a1*‐knockout uteri (Figure 6H). Notably, HCQ treatment did not significantly alter the elevated TIMP1 protein levels (Figure 6H).

Taken together, these findings show that HCQ treatment reduced embryo resorption and was accompanied by decreased uterine inflammation and collagen accumulation in *Col24a1*‐knockout mice.

### 3.8 Human Genetic Evidence Links *COL24A1* Variants to Severe Unexplained RPL

Building on our experimental findings showing that *Col24a1* deficiency promotes pregnancy loss in mice, we next explored the potential relevance of COL24A1 to human RPL. We screened whole‐exome sequencing (WES) data from patients in our institutional unexplained RPL cohort (n = 193) and identified a patient with severe RPL who had experienced 12 consecutive spontaneous early pregnancy losses with the same partner. The patient had no notable gynecological disorders, relevant family history, or other significant medical history. Comprehensive clinical evaluation identified no recognized cause of RPL, including immune disorders, thrombophilia, uterine structural abnormalities, or chromosomal abnormalities in either partner, and no consanguinity was reported.

We identified two rare heterozygous *COL24A1* variants (chr1:86581010 G>A and chr1:86453324 T>G) in this patient (Figure 7A) and independently confirmed both variants by Sanger sequencing (Figure 7B). Both were missense variants resulting in nonconservative amino acid substitutions, p.Arg515Trp and p.Asp775Ala. SIFT (http://sift.jcvi.org) and PolyPhen‐2 (http://genetics.bwh.harvard.edu/pph2) predicted both substitutions to be deleterious (Figure 7A). These substitutions involve physicochemical changes from polar (hydrophilic) to nonpolar (hydrophobic) residues that may affect protein structure or function (Figure S8A).

**FIGURE 7:**
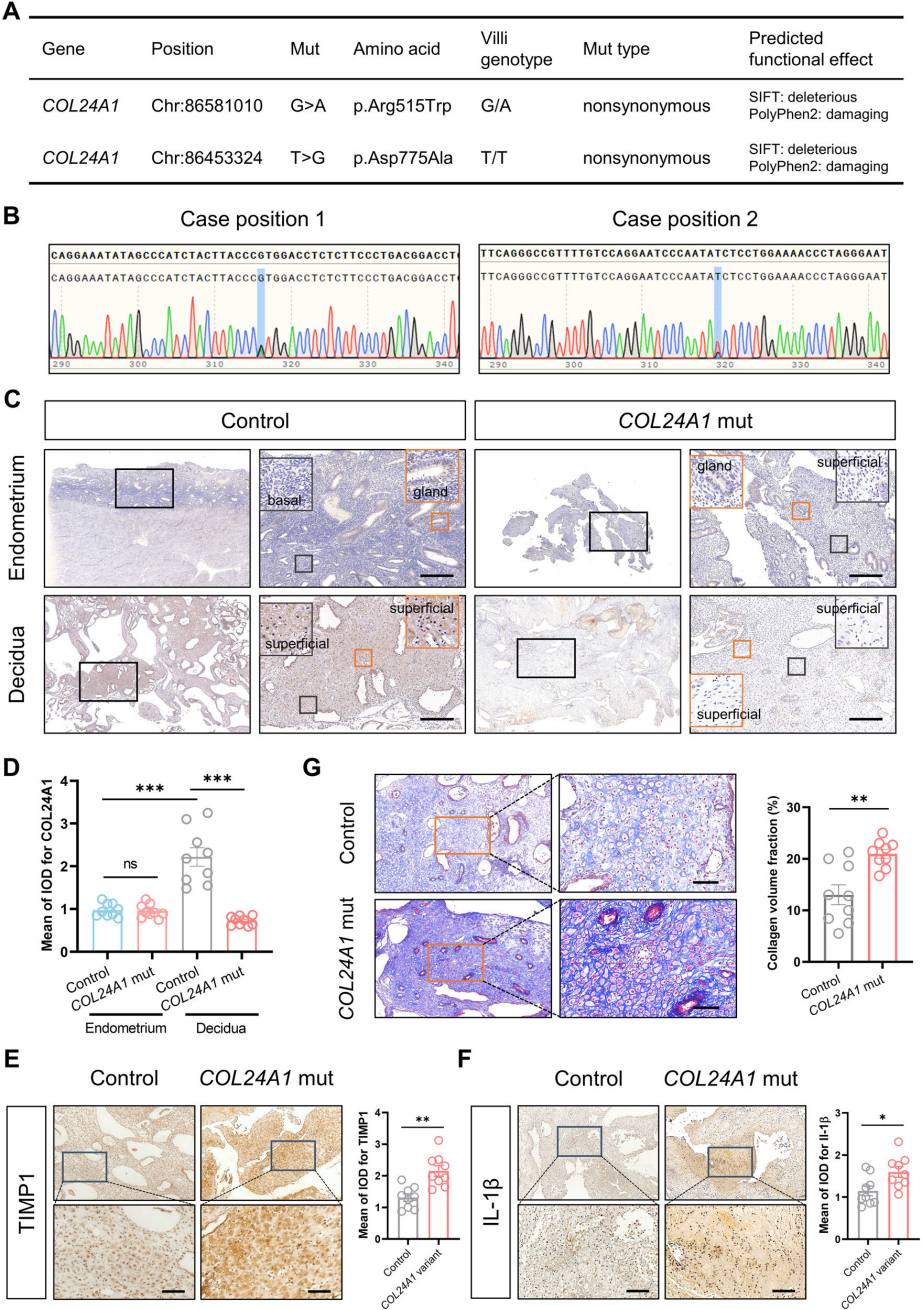
Identification and validation of heterozygous *COL24A1* variants in a patient with unexplained recurrent pregnancy loss (URPL). (A) Summary of the two rare heterozygous *COL24A1* variants identified in the patient, including genomic positions, nucleotide and amino acid substitutions, chorionic villus genotypes, variant types, and in silico functional predictions by SIFT and PolyPhen‐2. (B) Sanger sequencing validation of the two *COL24A1* variant loci identified by whole‐exome sequencing. (C, D) Representative immunohistochemistry (IHC) staining of COL24A1 (brown) and quantification in nonpregnant endometrium and pregnant decidua from control individuals and a patient carrying *COL24A1* variants. Control endometrial and decidual tissues were obtained from three independent individuals each; patient samples included endometrial tissue and miscarriage decidua from the patient carrying *COL24A1* variants. Nuclei were counterstained with hematoxylin (blue). Scale bars, 300 µm. (E, F) Representative IHC staining and quantification of TIMP1 (E; brown) and IL‐1β (F; brown) in decidual tissues from elective termination controls and the patient carrying *COL24A1* variants. Scale bars, 100 µm. (G) Representative Masson's trichrome staining of decidual tissues and quantification of collagen content in elective termination controls and the patient carrying *COL24A1* variants. Scale bars, 100 µm. Statistical analysis was performed by one‐way ANOVA and Tukey's multiple comparisons test (D), and unpaired Student's t‐test (E, F, G). Data are mean ± SEM. Differences were indicated as statistically significant at ^*^p <0.05, ^**^p < 0.01, and ^***^p <0.001.

We next examined COL24A1 expression in endometrial and decidual tissues from the patient and control subjects by immunohistochemistry. In controls, we observed relatively weak COL24A1 staining in the nonpregnant endometrium, including the superficial, glandular, and basal regions (Figure 7C,D, upper left half), whereas the superficial decidua showed stronger COL24A1 staining during pregnancy (Figure 7C,D, lower left half and Figure S8B). The patient showed a staining pattern comparable to controls in the nonpregnant endometrium, whereas the miscarriage decidua retained relatively weak COL24A1 staining compared with control decidua (Figure 7C,D, right panel). We also observed stronger TIMP1 and IL‐1β staining in the patient's decidual tissue than in control decidua (Figure 7E,F and Figure S8C). In addition, Masson's trichrome staining showed greater collagen deposition in the patient's decidual tissue than in control decidual tissue (Figure 7G).

Collectively, this severe unexplained RPL case carrying two rare *COL24A1* variants showed reduced decidual COL24A1 expression and increased collagen deposition. These tissue features paralleled key phenotypes observed in *Col24a1*‐deficient mice, providing cross‐species support for further investigation of COL24A1 as a potential susceptibility factor for RPL.

## 4 Discussion

This study identifies COL24A1, a uterine‐enriched fibril‐associated collagen, as a previously unrecognized regulator of pregnancy maintenance (Figure 8). Genetic ablation of *Col24a1* in mice caused a marked increase in embryo resorption at GD 13.5, accompanied by reduced fetal number and fetal weight, whereas estrous cyclicity and circulating sex hormone levels remained largely unchanged. These findings suggest that the pregnancy phenotype is unlikely to result primarily from endocrine dysfunction and instead point to abnormalities in the local uterine microenvironment. Supporting this view, histopathological and ultrastructural analyses of pregnant *Col24a1*‐deficient uteri revealed excessive collagen deposition together with inflammatory changes at the maternal‐fetal interface. In line with these structural abnormalities, bulk RNA‐seq and enzymatic analyses showed increased TIMP1 expression and reduced MMP2 activity, consistent with impaired extracellular matrix turnover. Single‐cell transcriptomic profiling further revealed expansion of a maladaptive Timp1‐high stromal cell subpopulation at the maternal‐fetal interface. This shift was accompanied by changes in extracellular matrix remodeling and stromal‐immune communication, suggesting that defective matrix remodeling may contribute to inflammatory dysregulation and pregnancy loss. Notably, analogous alterations were also observed in a patient with unexplained RPL, providing supportive human evidence for the potential relevance of COL24A1‐associated matrix abnormalities to RPL.

**FIGURE 8:**
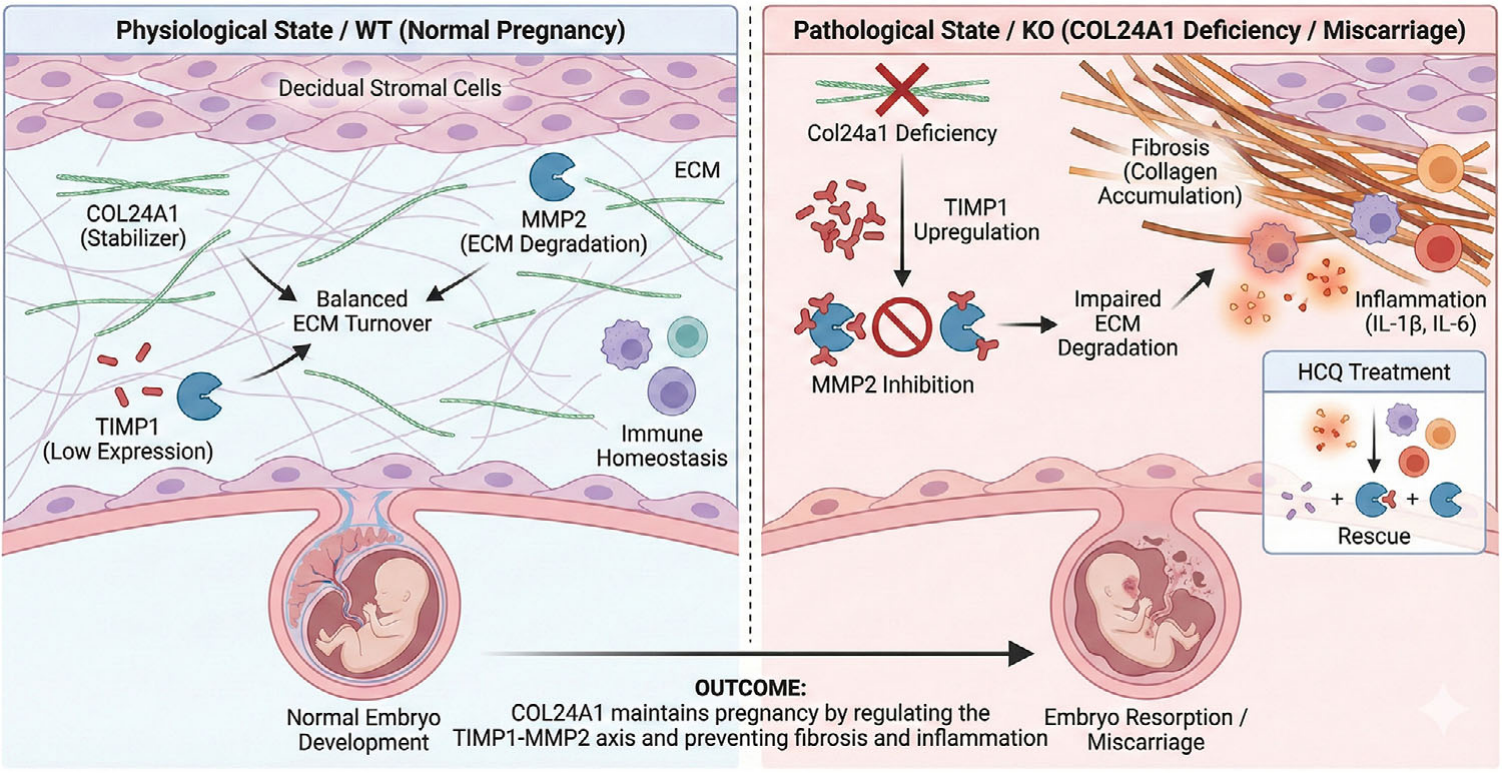
Graphical Abstract. Schematic overview of the proposed mechanism by which COL24A1 deficiency disrupts maternal‐fetal ECM‐immune homeostasis and contributes to pregnancy loss. (figure 8 was created with assistance from Gemini 3 Pro Image (Google; used February 2026) for visual illustration. The scientific content and mechanistic relationships were defined by the authors, and the AI‐assisted output was reviewed and revised by the authors for scientific accuracy. No primary experimental data or factual research images were generated or modified using AI.

Successful pregnancy depends on tightly regulated microenvironmental homeostasis at the maternal‐fetal interface. Within this niche, ECM remodeling, trophoblast invasion, vascular transformation, and immune adaptation must be coordinated spatially and temporally. Single‐cell and spatial multi‐omics studies indicate that such coordination emerges from precisely orchestrated interactions among decidual stromal cells, endothelial cells, diverse immune populations, and trophoblast lineages [4, 21, 22]. Disruption of these interaction networks is an important feature of pregnancy complications [23, 24], and disturbances in intercellular homeostasis may precede overt histopathological changes. Our findings extend this framework by identifying COL24A1 as a collagen‐associated regulator of maternal‐fetal interface homeostasis and as an upstream contributor to defective tissue remodeling that becomes evident under the increased remodeling demands of pregnancy. In our single‐cell analyses, *Col24a1* ablation increased the abundance of a maladaptive Timp1‐high stromal subpopulation and was associated with impaired ECM turnover and altered stromal‐immune signaling networks at the maternal‐fetal interface, potentially promoting a proinflammatory microenvironment. In turn, inflammatory signaling may further reinforce a TIMP1‐high state associated with reduced MMP2 activity and restricted matrix degradation [25, 26]. These changes may establish a self‐reinforcing cycle that shifts the interface toward a low‐proteolytic, fibrosis‐like remodeling state, promoting collagen persistence and progressive tissue dysfunction. Together, these findings integrate defective ECM turnover and altered stromal‐immune communication into a unified pathological axis at the maternal‐fetal interface and support the concept that ECM abnormalities may act as upstream contributors to immune dysregulation and pregnancy loss.

Pregnancy places the uterus under substantial remodeling demands. In this setting, COL24A1 appears to help maintain ECM homeostasis. *Col24a1* deletion produced few detectable changes in the nonpregnant uterus, whereas gestation revealed marked collagen accumulation and inflammatory activation. This pregnancy‐dependent phenotype also makes a primary endocrine mechanism less likely, as estrous cyclicity and circulating sex hormone levels remained largely unchanged. Instead, ECM degradation appears to be impaired in *Col24a1*‐deficient uteri, as indicated by increased TIMP1 expression and reduced MMP2 activity. Such an imbalance may favor collagen accumulation, potentially alter tissue stiffness and mechanical properties, and perturb the mechanical and biochemical cues required for coordinated cell migration, vascular remodeling, and immune homeostasis [12, 24, 27]. Thus, as remodeling demands increase during gestation, loss of COL24A1 may amplify these pathological changes, whereas they may remain largely subclinical under the comparatively lower remodeling demands of the nonpregnant state. Together, our findings suggest that COL24A1 is particularly important for maintaining uterine ECM homeostasis during pregnancy, when remodeling demands are substantially increased.

Our findings also have potential clinical and translational relevance. Identification of rare heterozygous *COL24A1* variants in a severe unexplained RPL case, together with reduced decidual COL24A1 expression and increased TIMP1 and fibrosis‐associated features, provides preliminary cross‐species concordance and supports *COL24A1* as a candidate susceptibility gene for a potential mechanistic subset of unexplained RPL—an area in which current guidance emphasizes the need for genetics‐informed and mechanism‐based stratification [1, 28]. However, we did not functionally validate the patient‐specific variants; variant‐level knock‐in models and complementary rescue experiments will therefore be important to establish causality and define allele‐specific mechanisms. In parallel, HCQ partially attenuated embryo resorption and improved fibrosis and inflammation‐related measures in the *Col24a1* knockout model, suggesting that the ECM‐immune coupling unmasked by COL24A1 deficiency may be amenable to pharmacological modulation. Recent studies have also suggested that HCQ may have therapeutic potential for alleviating intrauterine fetal growth restriction [29]. Nevertheless, clinical evidence supporting the efficacy of HCQ for RPL remains inconclusive [30, 31], underscoring the need for prospective studies incorporating mechanistic enrichment and biomarker‐guided stratification. Our findings provide a rationale for such an approach, and future studies could prioritize individuals exhibiting decidual ECM‐turnover defects together with a TIMP1‐high inflammatory signature.

Several limitations define important priorities for future investigation. First, the global knockout design cannot fully exclude contributions from extra‐uterine tissues or cell populations; uterine cell‐type‐specific deletion and rescue experiments will therefore be required to define the cellular origin of the phenotype. Second, the molecular mechanism linking *COL24A1* ablation to TIMP1 upregulation remains unresolved. Our single‐cell transcriptomic data nevertheless provide a framework for further mechanistic investigation by localizing TIMP1‐high states to defined cell populations and identifying inflammatory and stress‐response programs associated with TIMP1 expression. These analyses can prioritize candidate regulators and ligand‐receptor circuits for subsequent causal testing. Third, and most importantly, the current human genetic and phenotypic evidence derives from a single informative case. Larger independent cohorts will therefore be required to establish frequency of *COL24A1* variants, validate genotype‐phenotype associations, and determine their contribution to unexplained RPL.

## Author Contributions

H.C and C.W.C conceived and directed the study. T.W, L.L.X, W.Y.L, H.J.M, C.S.X, J.T, Z.R.W and P.X.L performed the primary experiments and analyzed the data. J.P.Z, J.G and Q.L.T provided discussion and assistance for the research. T.W and L.L.X wrote the manuscript. All authors read and approved the final manuscript.

## Funding

This work was supported by the National Key Research and Development Program of China (Grant No. 2019YFA0801403, to H.C.); the Beijing Health Promotion Association 2022 Young and Middle‐aged Doctors Research Grant (Grant No. BJHPA‐2022‐SHZHYXZHQNYJ‐LCH‐011, to H.C.); the Natural Science Foundation of Guangdong Province (Grant Nos. 2023A1515010515 and 2024A1515013264, to H.C.); the Sun Yat‐Sen Clinical Research 5010 Program (Grant No. SYS‐5010‐202410, to H.C.); Major Difficult and Complicated Diseases Integrated Traditional Chinese and Western Medicine Clinical Collaboration Project‐Threatened Abortion (Grant No. ZDYN‐2024‐A‐073, to H.C.); the National Natural Science Foundation of China (Grant No. 82500352, to T.W.), the China Postdoctoral Science Foundation (Grant No. 2025M781431, to T.W.); and the Sun Yat‐sen Pilot Scientific Research Fund (Grant No. YXQH202522, to T.W.; Grant No. YXQH202405, to A.Z.).

## Conflicts of Interest

The authors declare no conflicts of interest.

## Disclosures

The authors have nothing to report.

## Supporting information




**Supporting File**: advs78110‐sup‐0001‐SuppMat.docx.

## Data Availability

Data supporting the findings of this study are available from the corresponding author upon reasonable request. Detailed materials and methods are available in the Supplemental Material.
