# Supplementary material for: Structural Control of Stromal‐Immune Coupling by COL24A1 Underlies Pregnancy Maintenance
Source: Adv Sci (Weinh). 2026 Oct 4:e78110. Online ahead of print. doi: 10.1002/advs.78110 (PMC13634478; doi:10.1002/advs.78110)
Supplement: Supplementary file 1 [file ADVS-9999-e78110-s001.docx]

**SUPPLEMENTAL MATERIAL**

Supplemental Materials and methods

**Human specimens**

Human endometrial and decidual tissues were obtained from the Department of Obstetrics and Gynecology, Sun Yat-sen Memorial Hospital, Sun Yat-sen University (Guangzhou, China). All data and sample collection were in strict accordance with the ethics guidelines of Sun Yat-sen Memorial Hospital (2017-BA-04). Written informed consent was obtained from all participants before sample collection. All procedures were conducted in accordance with the Declaration of Helsinki.

Control specimens included endometrium from healthy women (n=3) and decidua from women with normal early intrauterine pregnancies undergoing elective termination (n=3). Pre-pregnancy endometrium, miscarriage decidua, and peripheral blood were collected from a patient with refractory unexplained RPL carrying rare compound heterozygous COL24A1 variants for tissue analysis and whole-exome sequencing. A standardized RPL evaluation excluded embryonic and parental chromosomal abnormalities, thrombophilia, uterine anatomical abnormalities, autoimmune etiologies, endocrine disorders, and infection. The patient’s age and clinical characteristics are provided in Supplementary Table 1.

**Experimental Animals**

All animal procedures were approved by the Institutional Animal Care and Use Committee (IACUC) of Sun Yat-sen University (SYSU-IACUC-2022-001356 and SYSU-IACUC-2022-001220). Additional details are provided in the sections below.

Mice were maintained on a C57BL/6 background under standard conditions (18-23°C; 40-60% humidity; 12-h light/dark cycle). Wild-type C57BL/6 males were obtained from the Laboratory Animal Center, Sun Yat-sen University (Guangzhou, China). *Col24a1* knockout mice (Cat. No. NM-KO-205550) were purchased from Shanghai Model Organisms Center, Inc. Homozygous knockouts were generated by intercrossing *Col24a1* heterozygotes. Mice were monitored for general health and showed no signs of illness or infection.

To determine the effect of *Col24a1* deletion on pregnancy loss, female *Col24a1* knockout mice and littermate WT females (10-14 weeks of age) were mated with WT C57BL/6 males. The day a vaginal plug was observed was designated gestational day (GD) 0.5. Pregnant females were euthanized at GD13.5, and uteri and conceptuses were collected for gross assessment. Implantation sites were scored as viable or resorbed, and fetal weights were measured. Tissues were fixed in 4% paraformaldehyde or stored at −80°C until analysis. Resorption rate (%) = [resorptions / (resorptions + viable fetuses)] × 100.

Hydroxychloroquine (HCQ) was administered to female *Col24a1* knockout mice in drinking water (HCQ Sulfate: 52 mg kg^−1^ BW/day). Treatment began 7 days before mating. Females were then mated with WT males as described above. HCQ administration continued through GD13.5. Mice were euthanized at GD13.5 for pregnancy phenotyping and tissue collection. Control *Col24a1* knockout females received drinking water without HCQ for the same duration.

**Conventional histopathology assay**

Uterine or decidual tissues were fixed in 4% paraformaldehyde (PFA) overnight, dehydrated through graded ethanol, cleared in xylene, paraffin-embedded, and sectioned at 5 μm. Sections were deparaffinized in xylene and rehydrated through graded ethanol to water. Hematoxylin and eosin (H&E) and Masson’s trichrome staining were performed according to the manufacturers’ protocols. For H&E staining, sections were processed using a commercial kit (Servicebio; Cat. No. G1005). Sections were stained with hematoxylin for 5 min and eosin for 2 min, washed, dehydrated, and mounted. Masson’s trichrome (Servicebio; Cat. No. G1006) was performed using commercial kits according to the manufacturers’ instructions. Representative images were selected from samples with values closest to the group mean. The collagen volume fraction (%) was quantified using Image-Pro Plus v6.0 (Media Cybernetics, Rockville, MD, USA). Each group included n = 3 or 6 uterine/decidual samples. Images were selected based on technical quality and representativeness of the group.

**Immunohistochemistry**

Paraffin-embedded sections were deparaffinized in xylene and rehydrated through graded ethanol. Antigen retrieval was performed in citrate buffer (pH 6.0) for 15 min using a microwave oven. Endogenous peroxidase activity was quenched with 3% hydrogen peroxide, followed by blocking with 3% bovine serum albumin (BSA). Sections were incubated overnight at 4°C with primary antibodies against COL24A1 (1:100; Biorbyt, orb513178), COL1A1 (1:200; Huabio, HA722517), IL-1β (1:200; Huabio, HA601036), or TIMP1 (1:200; Huabio, EM2001-03), or with matched rabbit/mouse IgG isotype controls. Sections were washed thoroughly to reduce nonspecific binding. Sections were incubated with an HRP-conjugated secondary antibody (ZSGB-BIO; Cat. No. PV-6000) for 30 min at room temperature. Signal was developed with 3,3′-diaminobenzidine (DAB) and sections were counterstained with hematoxylin (Solarbio; Cat. No. H8070). Images were acquired using a Leica DFC700T microscope (LAS AF software) and analyzed with Image-Pro Plus v6.0. Integrated optical density (IOD) was quantified for each section. Representative images were selected based on technical quality and consistency with the group-level quantification.

**Transmission electron microscopy (TEM)**

Uterine ultrastructure was examined by transmission electron microscopy (Tecnai G2 Spirit Twin with GATAN 832.10W; FEI, Czech Republic) using standard procedures. Briefly, ~1 mm^3^ uterine fragments were fixed in 2.5% glutaraldehyde and 2% paraformaldehyde in 0.1 mol/L phosphate buffer (pH 7.4), post-fixed with 1% osmium tetroxide, dehydrated, and embedded in epoxy resin. Ultrathin sections were cut, mounted on 200-mesh copper grids, and stained with uranyl acetate and lead citrate before imaging.

**Immunoblotting analysis**

Proteins were extracted from fresh mouse uterine tissues by homogenization in RIPA lysis buffer supplemented with a protease inhibitor cocktail. Lysates were centrifuged at 12,000 × g for 15 min at 4°C. Supernatants were collected, and protein concentrations were determined using a BCA assay (Thermo Fisher Scientific; Cat. No. 23225). Equal amounts of protein (50 μg) were separated by SDS-PAGE and transferred to PVDF membranes (Millipore). Signals were detected using horseradish peroxidase-conjugated secondary antibodies (Proteintech; Cat. Nos. SA00001-1 and SA00001-2) and enhanced chemiluminescence reagents (Tanon; Cat. No. 180-5001). Primary antibodies were as follows: COL1A1 (1:1000; Huabio, HA722517), COL3A1 (1:1000; Huabio, HA720050), IL-6 (1:1000; Huabio, R1412-2), IL-1β (1:1000; Huabio, HA601036), TIMP1 (1:1000; Huabio, EM2001-03), HSP90 (1:3000; Proteintech, 13171-1-AP), MMP2 (1:1000; Huabio, ET1606-4), MMP9 (1:3000; Huabio, ET1704-69), and β-actin (1:3000; Proteintech, 81115-1-RR). Band intensities were quantified by densitometry using ImageJ (NIH) and normalized to β-actin or HSP90. Representative blots were selected based on technical quality and consistency with the quantified results.

**Hydroxyproline assay**

The total collagen content in uterine tissue from mice at gestation day 13.5 was quantified using the hydroxyproline (HYP) content assay kit (Solarbio; Cat. No. BC0255) according to the manufacturer’s instructions.

**Cell culture**

Human endometrial stromal cells (HESCs) were cultured in DMEM/F12 medium supplemented with 10% FBS and 1% penicillin-streptomycin at 37°C in a humidified incubator with 5% CO₂. HESCs cultured under optimal conditions or after pretreatment were induced to undergo in vitro decidualization using DMEM/F12 medium containing 2% FBS and supplemented with estradiol (E2, 0.01 μM), medroxyprogesterone acetate (MPA, 1 μM), and 8-Bromo-cAMP sodium salt (8-Br-cAMP, 0.5 mM) for six days. The induction medium was changed every other day.

**RNA interference**

Small interfering RNAs (siRNAs) targeting the indicated genes were purchased from IGEbio (Guangzhou, China), including *COL24A1* (#1: UUGCUCAUCAUGAACACUGUA; #2: UUAAUUUGUGGAAGAAUAGAU). HESCs at approximately 30% confluence were transfected with 50 nM siRNA or negative control siRNA (NC) using jetPRIME® reagent (Polyplus-transfection; Cat. No. 101000046) according to the manufacturer’s instructions. Cells were harvested 36 h after transfection or after induction of in vitro decidualization. The efficiency of *COL24A1* knockdown was evaluated by RT-qPCR.

**Gelatin zymography assays**

Gelatin zymography was used to assess MMP2 and MMP9 activities in uterine tissues collected at gestational day (GD) 13.5. Gelatin-containing 8% SDS polyacrylamide gels were prepared using an MMP zymography kit (Applygen; Cat. No. P1700) according to the manufacturer’s instructions. After electrophoresis, gels were washed in Buffer A at room temperature for 36 h to remove SDS and renature enzymes. Gels were incubated in Buffer B at 37°C for 5 h to allow gelatin degradation. Gels were stained with Coomassie Brilliant Blue for 1 h, followed by three destaining washes, until clear lytic bands were visible. Band intensities were quantified using ImageJ (NIH).

**Real-time quantitative PCR**

Total RNA was extracted from tissues or cells according to the manufacturer’s instructions (GOONIE; Cat. No. 400-105) and reverse-transcribed using HiScript III RT SuperMix for qPCR (Vazyme; Cat. No. R323-01). Quantitative PCR was performed using SYBR-based detection on a QuantStudio 6 Flex system (Life Technologies). Gapdh or Hsp90 served as the reference gene, and relative expression was calculated using the 2^−ΔΔCt^ method. Primer sequences are listed in Supplementary Table 2.

**Serum measurement**

Mice were fasted overnight, and blood was collected from the retro-orbital plexus at the end of the experiment. Serum was separated by centrifugation. Serum estradiol (E2; Elabscience; Cat. No. E-OSEL-M0008), prostaglandin F2α (PGF2α; Elabscience; Cat. No. E-EL-M1360), and progesterone (PROG; Elabscience; Cat. No. E-OSEL-M0006) were quantified by enzyme-linked immunosorbent assay (ELISA).

**RNA sequencing (RNA-seq)**

Total RNA was extracted from uterine tissues collected at gestational day (GD) 13.5 from wild-type (WT) and *Col24a1* knockout females (n=3 per group). RNA concentration and integrity were assessed before library preparation. Libraries were sequenced on the MGIseq2000 platform (BGI, Shenzhen, China). Differential expression analysis was performed using edgeR, with DEGs defined as |log2 fold change| > 1 and adjusted P < 0.05. Gene Ontology enrichment analysis was performed using a Shiny web tool, with adjusted P < 0.05 considered significant.

**Single-cell RNA-seq and data processing**

Uterine tissues were collected from pregnant WT and *Col24a1* knockout mice at gestational day (GD) 5.5 and processed immediately as fresh specimens. Single-cell suspensions were generated using a standard dissociation workflow and assessed for cell quality before capture. Single cells were captured using the DNBelab C microfluidic system, followed by construction of standard RNA-seq libraries. Libraries were sequenced on the MGI DNBSEQ-T7RS platform in PE150 mode. All sample processing, library preparation, and sequencing were performed by Gene Denovo Biotechnology Co. (Guangzhou, China).

Raw sequencing data were processed using dnbc4tools for initial quality control, including removal of low-quality reads and summarization of sequencing output and quality metrics for each sample. Cells with fewer than 200 detected genes or more than 35,000 UMIs were excluded, and cells with more than 5% mitochondrial transcripts were discarded. After filtering, gene expression matrices were analyzed in Seurat (v4). Data were normalized and integrated across samples, with batch effects corrected using Harmony. Unsupervised clustering was performed using a graph-based approach, and clusters were visualized by uniform manifold approximation and projection (UMAP). Cell types were annotated using mLLMCelltype and then curated manually based on prior knowledge and canonical marker genes of the GD5.5 uterine microenvironment.

Differential expression analysis between *Col24a1* knockout and WT mice was performed within each annotated cell population. Significant differentially expressed genes were defined as those meeting *p* < 0.05 and |avg_log_2_FC| > 0.25. Functional interpretation was conducted using clusterProfiler for Gene Ontology (GO) enrichment and Kyoto Encyclopedia of Genes and Genomes (KEGG) pathway enrichment analyses on significant gene sets from each population; GO terms were grouped into biological process (BP), cellular component (CC), and molecular function (MF). Pseudotime trajectories were inferred using Monocle (v2.22.0). Gene expression and feature plots were generated using Seurat and ggplot2.

To infer ligand-receptor communication between stromal and immune cell subpopulations, we extracted expression matrices from the integrated single-cell dataset for each group and analyzed them using CellChat (v1.6.1). Changes in intercellular communication were identified by comparing predicted ligand-receptor interactions between groups. Only interactions with p < 0.05 were retained for downstream analyses.

RNA-seq and single-cell RNA-seq data have been deposited in the NCBI Gene Expression Omnibus database under accession number GSE326848.

**Hydroxychloroquine (HCQ)** **content assays**

HCQ concentrations in mouse serum and uterine tissue were quantified by LC-MS/MS (SCIEX Triple Quad^TM^ 7500; SCIEX) at the Instrumental Analysis and Research Center, Sun Yat-sen University, using an external calibration curve generated from HCQ reference standards. Samples were prepared on ice using prechilled, LC-MS-grade solvents. Serum (40 μL) or uterine tissue (~20 mg) was extracted with a prechilled acetonitrile/water mixture (3:1, v/v). Extracts were deproteinized by repeated centrifugation (15,000 rcf × g, 10 min, 4°C; ~3 cycles). Final supernatants were analyzed by LC-MS/MS, and concentrations were calculated by interpolation from the calibration curve.

Supplemental Tables

**Supplementary Table 1**. Clinical information for human samples.

| Participant | n | Specimen | Menstrual phase / gestational age | Age (years) | No. prior losses |
| --- | --- | --- | --- | --- | --- |
| Healthy controls | 3 | Endometrium | Proliferative phase | 34  35  25 | 0 |
| Normal early pregnancy controls | 3 | Decidua | 6 weeks  8 weeks  6 weeks | 35  26  44 | 0 |
| URPL case (**COL24A1** variants) | 1 | Endometrium (pre-pregnancy) | Proliferative phase | 40 | 12 |
| URPL case (**COL24A1** variants) | 1 | Decidua (miscarriage) | 6 weeks | 40 | 12 |

**Supplementary Table 2**. Primer sequences

| Gene name | Primer’s sequence 5' → 3' |
| --- | --- |
| Mouse-*Col24a1* | Forward: TGC CTA TAT TGA GTC ACC TCT CG  Reverse: TCT GTT GTT ATT CCG AAT GCT GA |
| Mouse-*Gapdh* | Forward: AGG TCG GTG TGA ACG GAT TTG  Reverse: GGG GTC GTT GAT GGC AAC A |
| Rat-*Col24a1* | Forward: CCT GGA GAA CAT GGC AAT CAA G  Reverse: TTT TCA CCT CTG GGT CCA GTT CC |
| Rat-*Hsp90* | Forward: TGA TGA TGA GGC TGA AGA AAA GGA  Reverse: CAG AAT TTT GCT TTG TTG CAG CAT |
| Human-*COL24A1* | Forward: TGC TGT GGA TCT CAC AAA CCA  Reverse: TTT GCC TCA CTG ATG CGA TGA |
| Human-*PRL* | Forward: TGA CCC TTC GAG ACC TGT TTG  Reverse: CTT GCT CCT TGT CTT CGG G |
| Human-*IGFBP1* | Forward: TTG GGA CGC CAT CAG TAC CTA  Reverse: TTG GCT AAA CTC TCT ACG ACT CT |
| Human-*FOXO1* | Forward: TCG TCA TAA TCT GTC CCT ACA CA  Reverse: CGG CTT CGG CTC TTA GCA AA |
| Human-*TIMP1* | Forward: CTT CTG CAA TTC CGA CCT CGT  Reverse: ACG CTG GTA TAA GGT GGT CTG |
| Human-*COL1A1* | Forward: GAG GGC CAA GAC GAA GAC ATC  Reverse: CAG ATC ACG TCA TCG CAC AAC |
| Human-*COL3A1* | Forward: GGA GCT GGC TAC TTC TCG C  Reverse: GGG AAC ATC CTC CTT CAA CAG |
| Human-*GAPDH* | Forward: GGA GCG AGA TCC CTC CAA AAT  Reverse: GGC TGT TGT CAT ACT TCT CAT GG |

Supplemental Figures

**Supplementary Figure 1.** **Validation of *Col24a1* knockout mice.** (A) Sanger sequencing of genotyping PCR amplicons confirming the targeted *Col24a1* allele in knockout mice. (B) Representative immunohistochemistry (IHC) images showing COL24A1 expression (brown) in the indicated organs from wild-type (WT) and *Col24a1* knockout mice. Scale bars, 50 μm.

**Supplementary Figure 2.** **Age-related pregnancy outcomes and uterine collagen and inflammatory marker expression in nonpregnant *Col24a1-KO* mice.** (A) Embryo resorption rates at GD13.5 in wild-type (WT) and *Col24a1-KO* dams at 10 and 28 weeks of age. (B) Number of embryos per dam at GD13.5 in WT and *Col24a1-KO* groups at 10 and 28 weeks of age (n = 20 mice at 10 weeks of age and n = 7 mice at 28 weeks of age). (C) Representative immunoblots and densitometric analyses of COL1a1, COL3a1, IL-1β, and IL-6 in uterine tissues from nonpregnant WT (n= 4) and *Col24a1-KO* (n= 4) mice; β-actin served as the loading control. Statistical analysis was performed by two-way ANOVA followed by Bonferroni's multiple comparisons test (B, C). Data are mean ± SEM. Differences were considered statistically significant at **p* < 0.05 and ****p* < 0.001.

**Supplementary Figure 3.** **Transcriptomic profiling of mouse uterine tissues.** (A) Schematic of the uterine RNA-sequencing (RNA-seq) workflow and analysis pipeline for wild-type (WT) and *Col24a1* knockout (*Col24a1-KO*) dams at GD13.5. (B) Gene set enrichment analysis (GSEA) of RNA-seq data from *Col24a1-KO* uteri compared with WT controls at GD13.5. (C) Gene Ontology (GO) enrichment analysis of genes significantly upregulated in *Col24a1-KO* uteri compared with WT controls. (D) Representative immunoblots and densitometric quantification of TIMP1 in uterine tissues from nonpregnant WT and *Col24a1-KO* mice. Statistical analysis was performed by non-parametric Mann-Whitney test (D). Data are mean ± SEM. Differences were indicated as statistically significant at **p* <0.05, ***p* <0.01 and ****p* <0.001.

**Supplementary Figure 4.** **Single-cell RNA-seq analysis of uteri from** **WT and *Col24a1-KO* mice.** (A) Experimental design. Uteri were harvested from WT and *Col24a1-KO* mice at gestational day 5.5 (GD5.5), with three uteri pooled per sample. (B) Bubble plot showing the expression of marker genes across 19 uterine cell types. (C) Bubble plot showing the expression of marker genes across four uterine stromal cell subpopulations. (D) Monocle analysis shows the ordering of stromal cells along pseudotime marked by uterine states. (E) Violin plots showing the expression of ECM organization- and integrin-mediated adhesion-related genes (*Col4a1*, *Dcn*, *Tnxb*, and *Itgb5*) in the Str_4 stromal population from WT and *Col24a1-KO* uteri.

**Supplementary Figure 5.** **The relative abundance of an inflammation-associated uNK cell subpopulation is increased in the uterus of *Col24a1-KO* mice during early pregnancy.** (A) UMAP plots of uNK cells showing five major clusters; colors indicate distinct clusters. (B) Bubble plot showing marker gene expression across the five uNK cell subpopulations. (C) UMAP plots of uNK cells from WT and *Col24a1-KO* uteri. (D) Proportions of uNK cell subpopulations in WT and *Col24a1-KO* uteri. (E) Violin plots showing pathway enrichment scores across uNK cell subpopulations. (F) Comparison of inflammatory response scores among uNK cell subpopulations.

**Supplementary Figure 6. *COL24A1*** **silencing impairs in vitro decidualization and alters ECM-associated gene expression.** (A) qPCR analysis of *COL24A1* mRNA expression in HESCs transfected with negative control siRNA (siNC) or *COL24A1*-targeting siRNA (*siCOL24A1*). (B) Relative mRNA levels of *PRL*, *IGFBP1*, and *FOXO1* in HESCs transfected with siNC or *siCOL24A1* at selected time points during in vitro decidualization (n = 3). (C-F) Relative mRNA levels of *COL24A1*, *TIMP1*, *COL1A1*, and *COL3A1*, respectively, in HESCs transfected with siNC or *siCOL24A1* at selected time points during in vitro decidualization (n = 3). Statistical analysis was performed using one-way ANOVA followed by Dunnett’s multiple comparisons test (A) and two-way ANOVA followed by Bonferroni's multiple comparisons test (B, C, D, E, F). Data are mean ± SEM. Differences were indicated as statistically significant at **p* <0.05, ***p* <0.01 and ****p* <0.001.

**Supplementary Figure 7. Effects of hydroxychloroquine sulfate on pregnancy outcomes in WT mice and uterine inflammation in *Col24a1-KO* mice.** (A) Schematic of the experimental design, including hydroxychloroquine sulfate (HCQ) administration and the experimental timeline. (B) Representative embryos and placentas at GD13.5. (C) Embryo resorption rate at GD13.5 in the indicated treatment groups of WT female mice (n = 6 dams per group). (D) Number of embryos per dam at GD13.5. (E) Representative IHC staining and quantification of IL-1β (brown) in uteri from *Col24a1-KO* female mice at GD13.5 (n = 6 mice per group). Nuclei were counterstained with hematoxylin (blue). Scale bars, 100 μm. Statistical analysis was performed by unpaired Student t test (C, D, E). Data are mean ± SEM. Differences were indicated as statistically significant at ***p* <0.01.

**Supplementary Figure 8.** **The URPL patient harbors two rare heterozygous variants in *COL24A1*.** (A) Schematic of the predicted aberrant COL24A1 protein structures associated with the two variants identified in the patient, compared with the wild-type protein. (B) Representative immunohistochemistry (IHC) image of decidual tissue from an elective termination control stained with rabbit IgG isotype control. (C) Representative IHC image of decidual tissue from an elective termination control stained with mouse IgG isotype control.
